# Adoption of an evidence-based colorectal cancer screening promotion program by community organizations serving Filipino Americans

**DOI:** 10.1186/1471-2458-14-246

**Published:** 2014-03-12

**Authors:** Annette E Maxwell, Leda L Danao, Reggie T Cayetano, Catherine M Crespi, Roshan Bastani

**Affiliations:** 1 Fielding School of Public Health and Jonsson Comprehensive Cancer Center, University of California Los Angeles, 650 Charles Young Dr. South A2-125 CHS, Box 956900, Los Angeles, CA 90095-6900, USA; 2 UCLA Kaiser Permanente Center for Health Equity, 650 Charles Young Dr. South A2-125 CHS, Box 956900, Los Angeles, CA 90095-6900, USA

**Keywords:** Lay health educator, Health promotion, Dissemination, Community organization, Churches, Adoption, Evidence-based intervention, Colorectal cancer screening

## Abstract

**Background:**

Filipino Americans have low rates of colorectal cancer (CRC) screening and high CRC mortality. To reduce this disparity, we conducted a dissemination trial in which we offered two levels of technical assistance to community organizations to disseminate an evidence-based CRC screening promotion program among their Filipino American members. This report describes the recruitment of organizations and adoption – the proportion and representativeness of organizations that decided to implement the program.

**Methods:**

During the recruitment phase, we completed organizational assessments with 44 community-based organizations (previous partners in research, organizations that were referred to us, or new organizations) to assess their eligibility to participate (having ≥ 150 Filipino American members age 50+). We compared organizational characteristics of organizations that did and did not adopt our CRC screening promotion program.

**Results:**

Twenty two of the 44 community organizations that completed the assessment adopted the CRC screening promotion program (50%). Adoption was highest among organizations that had previously partnered with us (11/14 = 79%) and among organizations that were referred to us by community partners (5/10 = 50%) and lowest among new organizations (6/20 = 30%). Few organizational differences were found between adopters and non-adopters.

**Conclusions:**

The high rate of adoption among organizations that were referred by community partners or had partnered with us in the past underscores the importance of community resources, community-academic relationships, and partnership in the dissemination process. However, the moderate rate of adoption among new organizations and the demands of completing documentation and assessments in our trial to advance dissemination research raise questions regarding the generalizability of study findings.

## Background

Filipino Americans have low rates of colorectal cancer (CRC) screening and high CRC mortality [1-3]. Community organizations such as service, social and civic organizations or churches may be uniquely positioned to convey health promotion messages to populations that do not receive routine health check-ups through the health care system [4]. We have previously developed an intervention to increase CRC screening among Filipino Americans in community settings and have demonstrated its efficacy in a randomized trial [5]. The intervention consisted of a small group educational session that was delivered by a trained Filipino health professional at community organizations, distribution of free Fecal Occult Blood Test (FOBT) kits and print materials, a reminder letter to participants, and a letter to their providers encouraging them to recommend CRC screening to this and all their eligible patients. Subsequently, a feasibility study demonstrated that this program can successfully be implemented in community organizations by trained community health advisors to promote CRC screening among members [6]. As a next step, we are studying the dissemination of this evidence-based intervention.

One of the first steps in launching a dissemination trial is to recruit organizations that are willing to adopt a specific intervention. Rogers defines adoption as “a decision to make full use of an innovation as the best course of action available” [7]. Part of the RE-AIM Evaluation Framework, adoption of an innovation can be estimated by assessing the proportion and representativeness of settings (such as community organizations) that adopt a given policy or program [8]. As described by Proctor et al. [9], adoption is one of several implementation outcomes that have to take place in order to achieve the desired long-term health outcome. Adoption is an important issue, since health programs or policies that are widely adopted in a variety of settings are likely to have a greater public health impact than those who are adopted by few organizations. Because few studies collect data from organizations that decide not to adopt a program, little is known about factors that are associated with adoption, especially the adoption of a health promotion program in community settings such as churches and other organizations that do not consider health promotion as their primary mission.

We conducted brief organizational assessments during the recruitment of community based organizations for an ongoing trial that aims to disseminate our evidence-based CRC screening program among Filipino Americans. In this paper, we compare responses to this organizational assessment from adopters and non-adopters to gain a better understanding of factors that may be associated with adoption of the program by community organizations. We also describe our efforts to recruit four different types of community based organizations and the resulting rates of adoption. A better understanding of organizations’ adoption of health promotion programs and factors that determine adoption would be useful for planning and conducting future community-based health promotion trials and would assist in the dissemination of evidence-based interventions through community venues.

## Methods

### Recruitment of organizations and screening for eligibility

From August 2010 to September 2011, we recruited organizations in Los Angeles and Orange counties that varied with respect to zip code level household income. Organizations were asked by a Filipino American member of the research team to complete a three page organizational survey to assess eligibility for participation in a study that aimed to increase colorectal cancer screening among Filipino Americans, because this group had lower screening rates and higher risk of dying from colorectal cancer than the general US population. Organizations were given the choice to answer survey questions face to face or by phone. One of the first questions asked about the size of their membership. Only organizations with at least 150 Filipino Americans age 50 and over were eligible to participate and were asked to complete the remaining questions about how long they had been in existence, number and type of programs/services offered, including health related programs (e.g., health fairs, walking groups, cooking classes), having an office and a directory of members (to distinguish formal versus informal organizations), having a Filipino ministry, a Filipino priest and Filipino language use during services (faith-based organizations only), their leadership structure (e.g., having an executive board, set of officers, committees, non-faith based organizations only) and interest in participating in the study. We attempted to recruit an equal balance of faith-based and non-faith based organizations, and of organizations that had participated in one of our prior CRC screening studies (previous partner organizations) and new organizations, using the following methods:

a. Previous partner organizations

Of the 45 organizations that had participated in our previous efficacy trial, 17 lacked the required membership and were not contacted. Phone calls to the remaining organizations determined that 12 had dissolved and 2 were not interested. The remaining 14 organizations that met the minimum membership requirement completed the organizational assessment.

b. New faith-based organizations

A list of 14 cities and neighborhoods in the Los Angeles and Orange counties with a large Filipino American population was compiled based on information from the US Census American Fact Finder (http://www.census.gov). Using the search words ‘Catholic churches’ and ‘Filipinos’, an online search identified 108 Catholic churches with Filipino American parishioners in these locations. Phone contact was attempted with the 65 of these with listed phone numbers. Of the 27 faith-based organizations that started the organizational assessment, 11 had the minimum membership needed and completed the survey.

c. New non faith-based organizations

A sample of 245 community based organizations was drawn from a directory of 457 CBOs listed in the 2004 Filipino Consumer Guide, the latest available version, published by the Asian Journal, a weekly Filipino-American newspaper in Los Angeles. Telephone messages and/or e-mails requesting information about their current status were sent to them, and 28 organizations responded. Nine organizations had the minimum membership needed and completed the survey.

d. Referred organizations

During the recruitment period, 11 organizations were referred to our study by community contacts. Ten of these had the minimum membership needed and completed the organizational assessment.

Organizations that completed the organizational assessment and were interested in promoting CRC screening among their members were invited to participate in “…a study that has the goal of disseminating CRC screening in the Filipino American community.” We reiterated that Filipino Americans have lower screening rates and a higher risk of dying from CRC than the general US population. All organizations were informed that participation would require the identification of 5 community health advisors who would be willing to attend a one day training, to recruit 10 Filipino Americans not up to date with CRC screening, and to implement the intervention (hold educational sessions, distribute free FOBT kit and print materials, remind individuals about screening, and mail a physician letter). Organizations were also informed that they would receive the following stipends for participation in the study: A stipend of $5,000, distributed over the 4 years of the study, was offered to each community organization for completing organizational assessments, for providing general support for the study, for supporting the community health advisors, and for paying an incentive of $20 to each participant who completed a baseline survey. Stipends were also offered to community health advisors for attending training and debriefing sessions ($150) and for completing research tasks, including obtaining informed consent from individual participants and HIPPA research authorization, conducting baseline surveys, and documenting their activities (up to $500 for each community health advisor).

### Theoretical considerations

Frambach and Schillewaert [10] distinguish *organizational factors related to adoption*, such as organization size and structure (the focus of this paper) and *factors related to adoption decisions made by an individual within an organization*, such as personal characteristics and beliefs (not assessed in our organizational survey). With respect to organizational factors, the argument has been made that larger organizations feel a greater need to adopt innovations to improve their performance. However, smaller organizations may be more flexible and innovative, which may result in the adoption of new programs. Similarly, it has been argued that organization structure (e.g., centralized versus less formalized) may influence adoption [10]. However, these organizational factors of adoption that are described in business and marketing research may not apply to community organizations and churches. Rogers [7] also proposed a number of constructs that influence adoption, including *program characteristics* such as compatibility, complexity and trialability. If a program requires a high level of expertise and a large amount of time to deliver, organizations are less likely to adopt it. Our program was thought to be compatible because it was developed specifically with and for the Filipino American community and had been tested in a variety of community settings [5]. Although it consisted of many different components (complexity), a pilot study had suggested that trained community members are able to implement it [6]. Organizations understood that they would be able to stop participation in the trial and dissemination of the program at any time (trialability). Organizations’ awareness of the problem addressed by the innovation also plays an important role for adoption [10]. In our study, it is likely that previous partner organizations that were engaged in our prior study promoting CRC screening were more aware about the problem of low CRC screening rates in the Filipino community than new organizations. A recent review found that constructs of organization size and structure, innovation fit with norms and values, and prior experience in adoption are considered in many theories related to innovation adoption [11].

### Statistical analysis

Organizations were classified as adopters if they agreed to participate in the study and to disseminate the CRC screening program and if they identified community health advisors who subsequently completed the one day training. Thus, we conceptualized adoption as the step prior to implementation. Organizational characteristics were compared between adopters and non-adopters using two sample t-tests for continuous variables and Fisher’s exact tests for categorical variables. The association of organizational characteristics with adoption was also assessed controlling for prior exposure to CRC studies, using logistic regression with adoption as the dependent variable and prior exposure and the characteristic of interest as independent variables. However, these adjusted analyses could not be conducted for faith-based organizations; because we did not survey faith-based organizations that had previously partnered with us but were not interested in adopting the program, there were no non-adopters with prior exposure.

This study was approved by the University of California, Los Angeles Institutional Review Board and is listed at clinicaltrials.gov (NCT01351220).

## Results

### Rates of adoption

Of 44 organizations that completed the organizational survey, 22 organizations (50%) adopted the program. The rate of adoption was similar for faith-based and non-faith-based organizations: 12/22 faith-based organizations (55%) and 10/22 non-faith-based organizations (45%) adopted the program. Adoption was highest among organizations that had previously partnered with us (11/14 = 79%) and among organizations that were referred to us by community partners (5/10 = 50%) and lowest among new organizations (6/20 = 30%). Many of the organizations that declined to adopt the program stated that they were too busy or not interested.

**Figure 1:**
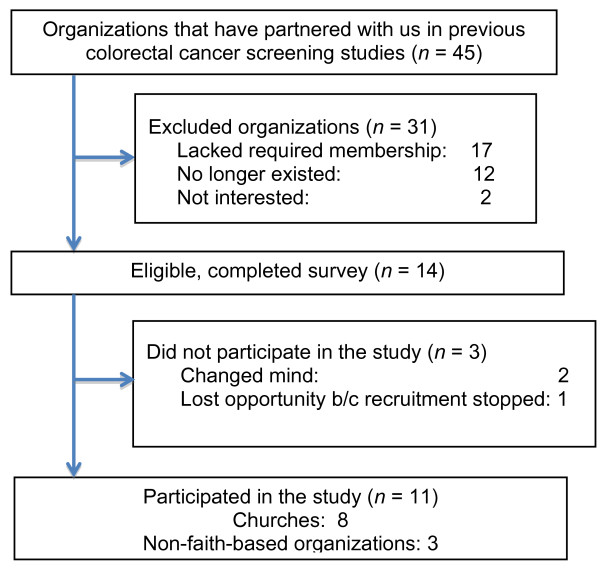
Flowchart summarizing recruitment of organizations that have partnered with us in previous colorectal cancer screening studies.

**Figure 2:**
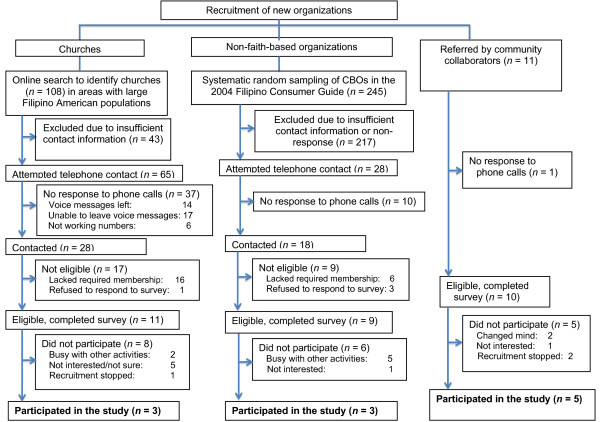
Flowchart summarizing recruitment of new organizations.

Details of the recruitment process are provided in Figures 1 and 2. As shown in Figure 1, of 14 eligible organizations that had previously partnered with us to promote CRC screening and had completed the organizational assessment, 11 (79%) adopted the program. As shown in Figure 2, of 11 new faith-based organizations that were eligible to participate and completed the organizational assessment, 3 (27%) adopted the program. Of 9 eligible new non-faith-based organizations, 3 (33%) adopted the program. Of 10 eligible organizations that were referred to us by community partners, 5 (50%) adopted the program.

### Comparison of adopters versus non-adopters

Table 1 compares *faith-based organizations* that adopted the program and those that did not adopt the program. In this small sample of organizations, no statistically significant differences emerged. Faith-based organizations that adopted the program tended to have more ongoing programs specifically for Filipino American parishioners (e.g., choir, bible study) than organizations that did not adopt the program (mean ± standard deviation: 6 ± 3 versus 4 ± 3, p = .11). Although a larger proportion of organizations that adopted the program tended to include Filipino language elements in church services than non-adopters (100% versus 60%, p = .09), adopters tended to be *less* likely to have a Filipino American priests than non-adopters (40% versus 90%, p = .06). Because we did not survey faith-based organizations that had previously partnered with us but were not interested in adopting the program, we had an unequal distribution of previous partners among adopters (8/12) and non-adopters (0/10; p < .002). No differences emerged with respect to zip code level household income (data not shown).

**Table 1:** Characteristics of faith-based organizations (N = 22) that did and did not adopt the program to increase CRC screening among their members

**Continuous variables**	**Adopted program**		**Did not adopt program**		
	**(n = 12)**		**(n = 10)**		
	**Mean ± SD**	**Min-max**	**Mean ± SD**	**Min-max**	**P**
Estimated number of Filipino-American members	3,422 ± 4,429	150-15,300	2,957 ± 2,566	600-8,400	.58
Number of health-related programs	2 ± 2	0-6	2 ± 2	0-5	.52
Number of years in operation	60 ± 32	5-107	50 ± 13	19-63	.36
Number of worship services per week^a^	14 ± 6	3-21	17 ± 4	12-24	.20
Number of Filipino community programs	6 ± 3	1-10	4 ± 3	0-10	.11
Number of priests^a^	4 ± 3	1-10	3 ± 1	2-5	.70
**Categorical variables**	**n/N**	**%**	**n/N**	**%**	**P**
Has a directory of members^b^	10/12	83	5/7	71	.60
Has an office	12/12	100	9/10	90	.46
Has a Filipino ministry^a^	9/10	90	8/10	80	.99
Offers regular Filipino mass^a^	6/10	60	4/10	40	.37
Includes Filipino language elements in services^a^	10/10	100	6/10	60	.09
Has Filipino priest(s)^a^	4/10	40	9/10	90	.06
Has a health program leader	5/12	42	2/10	20	.38
Exposure to CRC studies^c^	8/12	67	0/10	0	.002

^a^Analyses exclude two religious groups that do not have church services. ^b^Missing values for 3 non-adopters. ^c^Significantly different due to sampling: previous partner organizations who had been exposed to CRC studies but were not interested in adopting the program were not asked to complete the organizational survey.

P-values are from two-sample t tests for continuous variables and Fisher exact test for dichotomous variables. Estimated number of Filipino American members was log-transformed prior to the *t* test.

SD, standard deviation.

Table 2 compares *non-faith based community organizations* that adopted the program and those that did not adopt the program. The following two statistically significant differences emerged in bivariate analyses that remained significant after controlling for previous exposure to CRC studies: First, non-faith-based organizations that adopted the program reported having more health-related programs than those that did not adopt the program (mean + standard deviation: 3 ± 2 versus 0.5 ± 0.5, p < .05 in unadjusted and adjusted analyses). Second, organizations that adopted the program had been in existence fewer years than those who did not adopt the program (17 years ± 9 versus 26 years ± 7, p < .05 in unadjusted and adjusted analyses). Adopters also tended to have fewer Filipino American members (p = .05 in adjusted analysis) and fewer officers and board members than non-adopters, although the latter differences were not statistically significant.

**Table 2:** Characteristics of non faith-based organizations (N = 22) that did and did not adopt the program to increase CRC screening among their members

**Continuous variables**	**Adopted program**		**Did not adopt program**			
	**(n = 10)**		**(n = 12)**			
	**Mean ± SD**	**Min-max**	**Mean ± SD**	**Min-max**	**P**	**P, adjusted for previous exposure to CRC studies**^**a**^
Estimated number of Filipino-American members	209 ± 106	150-500	303 ± 151	158-648	.07	.05
Number of health-related programs	3 ± 2	0-7	0.5 ± 0.5	0-1	.02	.05
Number of years in operation	17 ± 9	3-30	26 ± 7	12-36	.02	.04
Number of programs/activities	4 ± 2	2-8	3 ± 1	2-5	.26	.31
Number of board members	7 ± 3	1-11	10 ± 6	0-23	.15	.17
Number of officers	8 ± 4	1-15	14 ± 9	5-35	.09	.11
Number of committees	2 ± 2	0-4	3 ± 3	0-10	.68	.58
**Categorical variables**	**n/N**	**%**	**n/N**	**%**	**P**	**P, adjusted for previous exposure to CRC studies**^**a**^
Has a directory/address book	9/10	90	11/12	92	.99	.85
Has an office	4/10	40	1/12	8	.14	.10
Previous exposure to CRC studies	3/10	30	3/12	25	.99	--

P-values are from two-sample t tests for continuous variables and Fisher exact test for dichotomous variables. Estimated number of Filipino American members was log-transformed prior to the *t* test.

SD, standard deviation.

^a^Logistic regression for the outcome of adoption, controlling for previous exposure to CRC studies (no exposure vs. previous exposure).

## Discussion

### Recruitment of organizations

Several studies have utilized community organizations for promoting health behaviors [5,12], but few have described their recruitment procedures and yields [13,14]. Our study illustrates the amount of effort needed to recruit organizations, although we did not keep logs of staff time and resources that were spent on recruitment. There is turnover among community organizations and some change location and telephone numbers, making them hard to contact. Other organizations that may have been interested in participating in our study were eliminated because they did not meet the eligibility criterion of having at least 150 Filipino American members age 50 and over. Few eligible organizations that were identified from a directory or an online search were receptive to a “cold” request to participate. Other studies have found moderate yields when using recruitment strategies that utilize online resources and directories [13,14]. Our yield of 27% to 33% of eligible organizations is similar to the 26% yield reported by Christensen et al., who recruited faith-based organizations from a church directory [13].

Recruitment was more efficient and yielded a higher adoption rate (79%) among organizations that had previously partnered with us. We already had information on their membership and were able to rule out organizations that did not meet the eligibility criterion, even without conducting an organizational survey. Factors that influenced their willingness to participate may include organizations’ positive experience in the prior study, a clear understanding of the planned intervention which was similar to that in the prior study, a greater awareness of the need to promote CRC screening in the Filipino community, a greater capacity or perceived self-efficacy to engage with the academic team, and/or personal relationships that existed with the study team. Anecdotal evidence from our study staff supports the importance of all of these “context” factors [15], although we were not able to assess them systematically among adopters and non-adopters. As in the study by Hippolyte et al. [14], a direct referral from a community partner was also a relatively effective recruitment strategy, which underscores the importance of community-academic relationships and partnership for engaging the community in health promotion efforts.

### Comparison of adopters versus non-adopters

The fact that no significant differences emerged between adopters and non-adopters among faith-based organizations may be due to the relatively small sample size and the relative homogeneity of the faith-based organizations that completed the organizational survey: all had at least 150 Filipino American members 50 years and over, and most had a Filipino American minister, an office and a directory of members. The finding that among adopters, 40% had a Filipino priest but among non-adopters 90% had a Filipino priest was unexpected. It could be that Filipino American priests did not want to adopt this program due to lower awareness about the problem of low CRC screening in their community, or they may be less willing than ministers from other racial/ethnic groups to volunteer their congregation for a research study. Future studies investigating adoption among faith-based organizations will likely need a larger and more heterogeneous sample.

For non-faith-based organizations, our results support the notion that newer but well established organizations with smaller membership and fewer board members and officers may be most likely to adopt a new program. Christensen et al. found that both being small (<100 eligible members) and being large (≥ 200 eligible members) predicted interest in their health promotion project that focused on dietary changes [13]. Additionally, adopters in our study had, on average, a larger number of pre-existing health-related programs than non-adopters. This may indicate both the organizations’ interest in implementing health-related programs and their capacity to do so. Most likely, organization size, interest and capacity are related and important determinants of adoption.

Although recruiting previous partner organizations and those that are referred to the research team by community partners may be more effective than attempting to recruit a variety of new organizations from directories and online sources, it raises the question of external validity. One may argue that recruiting from online resources and publicly available directories results in participation of organizations that more accurately reflect the overall pool of available organizations and thereby increases generalizability of findings. However, as Green and Nasser point out, “…the very act of agreeing to open the staff, patients, students, employees, or clients of an organization to the rigors of a controlled trial…makes that clinic, school, worksite, or other service-providing organization a special – possibly ungeneralizable – case” [16]. In our study, we required participating organizations to consent members for participation in the study, and to carefully document their activities so we would be able to describe the dissemination process and the effectiveness of the intervention among members. These attempts to achieve good internal validity posed a burden on the organizations and may have limited external validity, since only those organizations who felt capable of completing these activities adopted the intervention. Many community organizations engage in some type of health promotion, for example by participating in health fairs or by publishing healthy recipes in their newsletters. However, participation in our study imposed a prescribed number of activities and completion of assessment tools that exceeded these “sporadic” dissemination activities. Future dissemination trials will need to find a balance between studying the dissemination process and letting dissemination “happen” with minimal monitoring.

### Limitations

Organizations and community health advisors received compensation for performing research tasks, including consenting participants, administering baseline interviews and documenting their efforts. The fact that organizations were informed about these stipends along with other requirements for participation may have increased rates of adoption. Community organizations often have limited financial resources and many organizations may not be able to adopt, implement and maintain health promotion efforts without compensation. This raises the question of the extent to which community organizations such as churches that focus on non-health related activities can engage in the dissemination of health promotion programs and what resources they need to maintain their efforts. The study was limited to organizations in the Los Angeles area that served a large number of Filipino Americans 50 years and older. Findings may not apply to other organizations. In addition, many potentially eligible organizations declined to complete the organizational assessment. Had we been able to survey more of these organizations, we may have found more differences between adopters and non-adopters and lower rates of adoption.

## Conclusions

Organizations that were referred by community partners or had partnered with us in the past had the highest rate of adoption, underscoring the importance of community resources, community-academic relationships, and partnership in the dissemination process. However, the moderate rate of adoption among new organizations and the demands of completing documentation and assessments in our trial to advance dissemination research raise questions regarding the generalizability of study findings.

## Competing interests

The authors declare that they have no competing interests.

## Authors’ contributions

AEM conceived of the study, took responsibility for the overall conduct of the study, conceptualized the paper and drafted the manuscript. LLD and RTC carried out the study activities and provided feedback on the manuscript. CMC and RB participated in the design of the study and the conceptualization of the manuscript. CMC oversaw the statistical analysis. All authors read and approved the final manuscript.

## Pre-publication history

The pre-publication history for this paper can be accessed here:

http://www.biomedcentral.com/1471-2458/14/246/prepub
